# Cerebrospinal fluid biomarkers in Parkinson’s disease with freezing of gait: an exploratory analysis

**DOI:** 10.1038/s41531-021-00247-x

**Published:** 2021-11-29

**Authors:** J. M. Hatcher-Martin, J. L. McKay, A. F. Pybus, B. Sommerfeld, J. C. Howell, F. C. Goldstein, L. Wood, W. T. Hu, S. A. Factor

**Affiliations:** 1 Jean & Paul Amos PD & Movement Disorders Program, Department of Neurology, Emory University, Atlanta, GA USA; 2 Department of Biomedical Informatics, Emory University, Atlanta, GA USA; 3 Wallace H. Coulter Department of Biomedical Engineering, Georgia Tech and Emory University, Atlanta, GA USA; 4 Department of Mechanical Engineering, Georgia Tech, Atlanta, GA USA; 5 Neurology Consultants of Dallas, Dallas, TX USA; 6 Neuropsychology Program, Department of Neurology, Emory University, Atlanta, GA USA; 7 Alzheimer’s Disease Research Center, Emory University and Cognitive Neurology Division, Rutgers-Robert Wood Johnson Medical School and Institute for Health, Health Care Policy, and Aging Research, Atlanta, GA USA; 8 Present Address: Department of Neurology, SOC Telemed, Atlanta, GA USA

**Keywords:** Parkinson's disease, Parkinson's disease

## Abstract

We explore the association between three Alzheimer’s disease-related and ten inflammation-related CSF markers and freezing of gait (FOG) in patients with Parkinson’s disease (PD). The study population includes PD patients with FOG (PD-FOG, *N* = 12), without FOG (PD-NoFOG, *N* = 19), and healthy controls (HC, *N* = 12). Age and PD duration are not significantly different between groups. After adjusting for covariates and multiple comparisons, the anti-inflammatory marker, fractalkine, is significantly decreased in the PD groups compared to HC (*P* = 0.002), and further decreased in PD-FOG compared to PD-NoFOG (*P* = 0.007). The Alzheimer’s disease-related protein, Aβ42, is increased in PD-FOG compared to PD-NoFOG and HC (*P* = 0.001). Group differences obtained in individual biomarker analyses are confirmed with multivariate discriminant partial least squares regression (*P* < 0.001). High levels of Aβ42 in PD-FOG patients supports an increase over time from early to advanced state. Low levels of fractalkine might suggest anti-inflammatory effect. These findings warrant replication.

## Introduction

Freezing of gait (FOG) is characterized by arrests of stepping when initiating gait, turning, and walking straight ahead and patients describe it as their feet being “glued” to the floor^1^. While it is a well-known feature of Parkinson’s disease (PD), it has been reported in other parkinsonian disorders as well^2^. FOG frequency in PD patients increases with disease duration and occurs in >60% of patients with ≥10 years of disease^3^. It is a gait symptom complex that has potentially grave consequences^1,4^ as it is unpredictable in character, a leading cause of falls with injury^5^, and results in loss of independence and social isolation. Treatment options are limited^1,6^. Although FOG is considered to be a cardinal feature of PD, it appears to develop and/or progress independently of the other cardinal motor features^7^. It is associated with specific clinical risk factors (longer disease duration, psychotic symptoms, and absence of tremor), is associated with cognitive change, and is thought to be caused by specific as yet unknown pathology^8–10^. The pathophysiology of FOG remains poorly understood. The literature shows great variability in findings related to physiological and imaging research, as well as motor and non-motor correlates and therapeutic response to various treatment modalities^9,11–13^ suggesting it possibly relates to a multimodal circuit change.

There has been little in the way of biofluid research in FOG. One study of data from the Parkinson Progression Marker Initiative study (PPMI) showed that low CSF β-amyloid42 (Aβ42) levels in early PD predicted incident FOG within the first few years after diagnosis^14^ using measures from the Movement Disorder Society-Unified Parkinson’s disease Rating Scale (MDS-UPDRS) and this has been supported by increased neocortical deposition of β-amyloid in the brain with imaging studies^15^. However, these CSF data have been reported only for the first 3 years after disease onset. In this study, we present initial results of CSF analysis in more advanced PD patients with and without FOG (average disease duration ~10 years) and age-matched healthy controls (HC). We also present results describing variation in CSF markers over a wide range of disease duration (onset–23 years).

## Results

### Participants

CSF samples were collected from PD patients with FOG (PD-FOG) (*N* = 12), PD patients without FOG (PD-NoFOG) (*N* = 19), and HC (*N* = 12) for analysis. Clinical and demographic characteristics are presented in Table 1. No significant differences in age, PD duration, or ON state MDS-UPDRS-III scores were identified between groups. However, the HC group included more females than either of the PD groups (67, 37, and 8% in the HC, PD-NoFOG, and PD-FOG groups, respectively; *P* < 0.01), MoCA score also varied across groups (*P* = 0.05), with the lowest scores observed in PD-FOG; and individuals in the PD-FOG group were on significantly higher doses of levodopa equivalent daily dose (LEDD, *P* < 0.01).

**Table 1:** Demographic and clinical features of the study sample.

	HC	PD-NoFOG	PD-FOG
*N*	12	19	12
Age, y	74.4 ± 10.0	70.4 ± 10.1	70.7 ± 8.3
Sex*			
Female	8 (67%)	7 (37%)	1 (8%)
Male	4 (33%)	12 (63%)	11 (92%)
MoCA score^†^	28.3 ± 1.6^a^	26.9 ± 3.5^b^	24.3 ± 4.2
PD duration, y		9.6 ± 4.2^c^	11.5 ± 5.7
MDS-UPDRS-III		23.8 ± 13.4^a^	19.1 ± 9.1
LEDD, mg^‡^		662 ± 358^d^	1598 ± 865
FOG duration, y			4.4 ± 3.7
NFOG-Q			21.0 ± 4.8

Data were presented as mean ± standard deviation or as *N* (%).

*MoCA* Montreal cognitive assessment, *MDS-UPDRS-III* unified Parkinson’s disease rating scale, movement disorders society revision, section III (“ON medication state), *LEDD* levodopa equivalent daily dose, *NFOG-Q* new freezing of gait questionnaire.

*,†,‡ Significant difference between groups: *P < 0.01, chi-squared test, †*P* = 0.05, ANOVA; ‡*P* < 0.01, *t*-test.

^a^*N* = 7.

^b^*N* = 14.

^c^*N* = 18.

^d^*N* = 15.

### Variation in CSF marker expression with PD and FOG

**Fig. 1:**
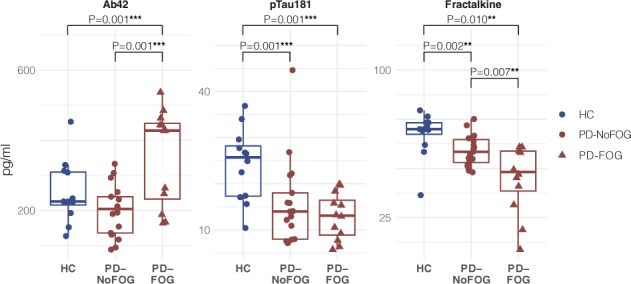
CSF biomarker levels. Box plots depicting expression of the CSF markers Aβ42, p-Tau_181_, and Fractalkine across study groups. Boxes and horizontal lines depict ranges Q1–Q3 and median values of the expression of each marker, respectively. HC healthy control, PD-NoFOG PD without FOG, PD-FOG PD with FOG. *P* values reflect multivariate linear models controlled for sex, PD duration, and false discovery rate. **P ≤ 0.01, Wald tests. ††*P* = 0.007, post hoc *F*-test between PD-NoFOG and PD-FOG controlling for sex and PD duration.

Levels of CSF markers that varied across groups are summarized in Table 2 and presented graphically in Fig. 1. Initial univariate ANOVAs identified five CSF markers with significant variation across groups: Aβ42, p-Tau_181_, fractalkine, MCP-1, and TGFα. Univariate ANOVA results for all CSF markers entered into analyses are summarized in Supplementary Table 2. After correction for false discovery rate, Aβ42, p-Tau_181_, and fractalkine remained statistically significant and were entered into multivariate linear models adjusted for disease duration, sex, and multiple comparisons (Table 2). Adjusted models showed that the anti-inflammatory marker, fractalkine, was significantly decreased in PD-NoFOG and PD-FOG groups compared to HC (≈23%, *F*_1,37_ = 12.6, *P* = 0.002, adjusted for sex and duration), and further decreased in PD-FOG compared to PD-NoFOG (≈24%, *F*_1,27_ = 8.7, *P* = 0.007). The AD-related protein, Aβ42, was significantly increased in PD-FOG compared to the other groups (vs. PD-NoFOG, ≈44%; vs. HC, ≈30%) (*F*_1,24_ = 13.9, *P* = 0.001, adjusted for sex and duration. p-Tau_181_ was also significantly decreased in both PD groups compared to HC (≈40%, *F*_1,35_ = 24.7, *P* < 0.001, adjusted). All statistically significant *P* values remained so after correction for multiple comparisons using a Benjamini–Hochberg procedure. No statistically significant effects of sex or PD duration were identified in multivariate linear models.

**Table 2:** Differential expression of CSF biomarkers across study groups.

Biomarker	HC	PD-NoFOG	PD-FOG
*N*	12	19	12
Aβ42^*^	250.6 ± 88.3	198.9 ± 72.8^a^	354.7 ± 137.3
p-Tau_181_^†^	24.1 ± 7.8	15.7 ± 9.4^a^	12.9 ± 5.0
Fractalkine*^,†^	67.5 ± 11.3	59.0 ± 7.8	44.9 ± 17.3

Wald test, adjusted for sex, disease duration, false discovery rate.

All values are expressed as mean ± SD pg/ml.

^*^Significant difference (*P* < 0.01) between PD-FOG and other groups.

†Significant difference (*P* < 0.01) between HC and other groups.

^a^*N* = 17.

To protect against violations of normality due to the small sample size, and potential confounding by sex, *P* values from initial ANOVAs were compared to those (1) from nonparametric Kruskal–Wallis tests applied post hoc and (2) from stratified ANOVAs conducted only among males post hoc. In both cases, *P* values for Aβ42, p-Tau_181_, and fractalkine remained statistically significant (Supplementary Table 2).

### Discriminant partial least squares regression analysis of biomarker variation across groups

**Fig. 2:**
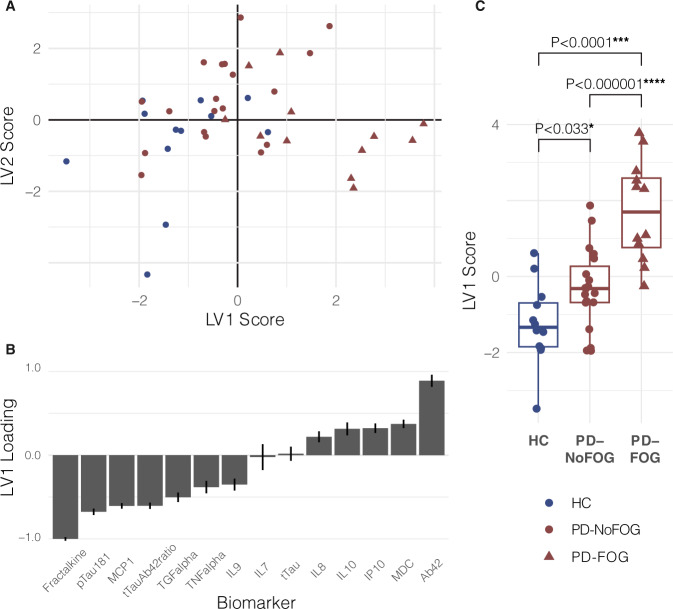
D-PLSR Results. **A** All biomarker data were used to identify two latent variables, LV1 and LV2. Each dot represents a study participant plotted in latent variable space. **B** The strongest loadings of LV1 (at each end) were in biomarkers identified as significantly varying with PD or FOG in single biomarker analyses. **C** LV1 expression varied significantly across study groups. *P* values reflect post hoc pairwise *t*-tests conducted subsequent to omnibus ANOVA. A type-I error was controlled with Holm–Bonferroni procedure.

Group differences in biomarker expression described above were confirmed with discriminant partial least squares regression (DPLS-R). DPLS-R identified two latent variables, LV1 and LV2, within the biomarker data that explained the majority of the variation in the biomarker expression and that were able to differentiate participants according to study group (Fig. 2A). The first latent variable LV1 consisted of a profile of CSF biomarkers weighted according to how they were differentially expressed in HC, PD-NoFOG, and PD-FOG samples. Consistent with the results of analyses of individual CSF biomarkers, the strongest loadings in LV1 were identified for Aβ42, p-Tau_181_, and fractalkine (Fig. 2B). ANOVA and post hoc tests identified significant differences in LV1 loading across groups (*P* < 0.001) and differences between each pair of groups (HC vs. PD-NoFOG, *P* < 0.05; HC vs. PD-FOG, *P* < 0.0001; PD-NoFOG vs. PD-FOG, *P* < 0.000001) (Fig. 2C).

### Variation in CSF markers with disease duration

**Fig. 3:**
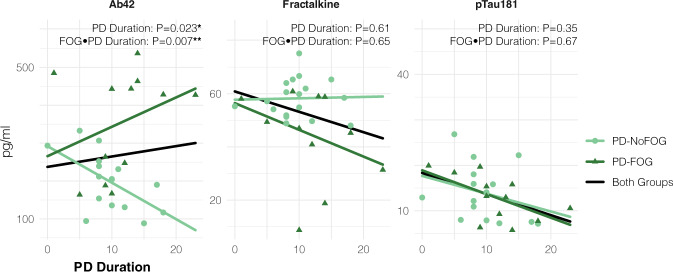
Association between CSF markers (Aβ42, fractalkine, and p-Tau_181_) and disease duration, stratified by presence of FOG. Dark and Light Green lines represent separate linear regressions of marker level onto PD duration for PD-FOG and PD-NoFOG, respectively. Gray lines represent linear regressions for both groups combined. *P* values reflect multivariate linear models with terms for age, sex, FOG, PD duration, and interaction between FOG and PD duration.

Associations between each biomarker identified as statistically significant in multivariate analyses and PD duration is shown in Fig. 3. There was a significant linear relationship (*P* = 0.02) between Aβ42 expression and PD duration with significant (*P* < 0.01) interaction between the PD-FOG and PD-NoFOG groups in multivariate models controlling for age and sex. Aβ42 was positively associated with PD duration in the PD-FOG group but negatively associated with PD duration in the PD-NoFOG group. No other statistically significant associations were identified. Visual inspection of plots indicated some evidence of interaction between groups for fractalkine, in which the PD-FOG group decreased with increasing duration whereas the PD-NoFOG group increased somewhat, and no evidence of interaction for p-Tau_181_, in which both groups exhibited a decreasing relationship with PD duration.

## Discussion

In this study we examined, in CSF, three AD-related markers and ten inflammation-related proteins in HC and in PD patients with and without FOG. Among those with PD and FOG—which was carefully characterized clinically and in our motion capture lab—multivariate models showed that Aβ42 was elevated compared to PD-NoFOG cases and to HC. We also found that the anti-inflammatory protein fractalkine was lower in PD-FOG vs PD-NoFOG and HC. While we identified reduced CSF p-tau among both PD groups vs. HC, which was consistent with multiple studies^16^, the changes in Aβ42 and fractalkine were unexpected and in the opposite direction than those seen in comparisons of AD and HC.

Aβ42 is a CSF marker that is low in AD^17^ and represents the first marker change^18^ that predicts the development of AD pathology. The low levels in AD may reflect increased accumulation in the brain, enhanced clearance, or some other yet unknown mechanisms^19^. Normally, there is a diurnal pattern of CSF amyloid, higher during wakefulness and lower during sleep. This diminishes with age and even more so in AD and relates to sleep disruption which is caused by Aβ42 aggregation as seen in animal models^20^. In PD, much of the data on CSF markers come from early-stage patients in PPMI. Aβ42 CSF levels in early PD (within 2 years of symptom onset) have been shown to be modestly lower than HC (≈9%, in baseline measures)^21–23^. At levels below a critical value, Aβ42 levels appear to be associated with earlier development and faster decline of cognitive function in PD patients^24^ especially in those with REM sleep behavior disorder^25^ or the *APOE* ε4 allele^26^. However, in those with CSF Aβ42 levels closer to typical values, relationships between CSF Aβ42 and progression are less clear^27^. The association of low baseline CSF Aβ42 with early dementia in PD has been shown by other investigators as well^28^. One study suggested an association of low baseline levels to worse motor scores and, in particular, postural instability gait disorder (PIGD) sub scores^23^ although such findings have not been consistently seen. The neuropathological correlates of lower CSF Aβ42 in PD also remain unclear. Whether those with low levels of CSF Aβ42 represent a subset of PD patients with coincident AD amyloid accumulation or altered amyloid metabolism in the brain remains to be seen, although previous in vitro animal and clinical cohort studies have suggested the increasingly important role of AD pathology in the development of PD Dementia^29^.

Our results suggest that the associations between lower CSF Aβ42 and incident FOG that hold in newly diagnosed PD patients^14^ may not generalize to older or more advanced patients. FOG is also associated with cognitive decline, particularly executive dysfunction^8–10^ which, as cited, is associated with lower Aβ42. Our results in this study would appear to be in opposition to these findings, as we demonstrated higher CSF Aβ42 levels among the PD-FOG group. Our CSF samples were taken in much later stages of disease than are currently available in studies like PPMI; the mean duration of the disease was 10.4 (±4.8) years and the longest duration in the sample was 23 years. Commensurate with their increased disease duration, these individuals were older than those in the most recent reports from PPMI (71.6 years vs. 61.4 years in PPMI)^23^. Additionally, among the FOG group here, there was no evidence of substantial cognitive dysfunction—the average MoCA score was 26.9 ± 3.5 in the PD-NoFOG group and 24.3 ± 4.2 in the PD-FOG group. This could suggest an increase in CSF levels of Aβ42 in the ensuing time frame, perhaps due to increased amyloid production—or decreased sequestration in the brain—particularly in the PD-FOG group. Our cross-sectional analysis of the relation between CSF Aβ42 and duration of disease in the PD-FOG and PD-NoFOG would also suggest that this increase is specific in PD to those developing FOG, as we found a statistically significant interaction in the relationship between CSF Aβ42 and disease duration: Aβ42 increased with increasing duration in the PD-FOG group but decreased with increasing duration in the PD-NoFOG group. Such a pattern is generally not seen with AD. Although it has been shown in AD that longitudinal changes of CSF marker levels vary in distinct populations of subjects depending on underlying pathology^30^. There is limited data on longitudinal changes in levels over time in PD. Data from the PPMI study demonstrated an increase in Aβ42 over 6 and 12 months of follow-up in PD and HC groups which reached significance only at the 1-year mark compared to baseline (a 4% increase)^31^. This correlated with age in both groups and disease duration in the PD group. They did not examine subgroups such as those with FOG. Irwin et al. more recently examined AD markers in PD and HC in a larger number of patients from the PPMI cohort and with a different assay from the prior assessment, this time followed up to 3 years. In longitudinal analysis they found that the PD cohort had a modestly greater decline in CSF Aβ42 (mean difference = −41.83 pg/mL; *p* = 0.03) and CSF p-tau (mean difference = −0.38 pg/mL; *p* = 0.03) at year 3 compared with HC^23^. These findings are consistent with our observation for PD-NoFOG. It is, therefore, possible that not only the baseline level but the pattern of change of Aβ42 over time may be predictive of the development of clinical outcomes, with increasing levels later in FOG. For example, in early PD, Kim et al.^14^ recently showed moderately reduced (≈8%) CSF Aβ42 levels among patients who develop FOG within the first 4 years. In linear trends estimated from our small dataset, a similar relationship is observed over the first ≈3 years, but after this point FOG is associated with increased, rather than decreased, CSF Aβ42 (Fig. 2). Further longitudinal observations in PPMI subjects or other studies are needed to define the extent to which the prognostic performance of CSF markers varies with age or disease duration. It should be noted that high CSF Aβ42 levels is not unique to this PD-FOG cohort. It also appears in other scenarios including; slow-wave sleep disruption^32^, narcolepsy with cataplexy (especially those with normal CSF hypocretin-1 concentrations)^33^, particular gene polymorphisms in AD^34^, late-life depression^35,36^, and traumatic brain injury^37^. It would be important in the future to examine for these diagnoses.

The existing evidence does not allow for definite conclusions regarding the role of fractalkine in PD pathophysiology, but our results suggest an anti-inflammatory effect with lower levels being associated with PD-FOG and with PD-NoFOG compared to HC. Fractalkine is a neuroimmune regulatory protein produced mainly by neurons and exists in a native, membrane, and soluble forms, each eliciting different cytokine responses from immune cells in the central and peripheral nervous systems. The soluble form, which is measured in this study, has a signaling function specifically through the G-protein-coupled CX3CR1 receptor that resides on microglia^38^. Fractalkine is known to have an anti-inflammatory function under some circumstances as signaling contributes to suppressing microglial activation and maintaining the microglia surveillance phase^39^. As part of this function, it reduces the overproduction of proinflammatory molecules such as inducible nitric oxide synthase, interleukin (IL)-1β, (TNFα), and IL-6 generated by microglia^38,39^. However, in rodent toxin models it has been shown that the exact effects greatly depend on the isoform type (soluble or membrane-bound), animal model (mice or rats, toxin- or proteinopathy-induced), route of toxin administration, time course, specific brain region (striatum, substantia nigra), and cell type^39^. The same is true with regard to α-synuclein models, inflammatory response type depends on the form of α-synuclein (overexpressed wild-type or A53T-mutated form), the specific isoform of fractalkine, and the experimental protocol^39^. Whenever neuroprotective, the soluble, and not the membrane-bound form of fractalkine seems to be responsible for its beneficial role^39^. Similar variation in results in AD models has been reported^40^. In one study, in transgenic mouse models of AD (amyloid precursor protein/-presenilin1 and CX3CR1−/−), fractalkine brought about a decrease in amyloid burden^41^. Alzheimer’s disease studies of fractalkine levels have demonstrated conflicting results but recently a reduction in soluble FKN was reported in the cerebrospinal fluid as seen in our PD cohort^40^

One previous CSF study in PD found no difference in fractalkine levels between HC and PD, but it is not clear if participants with FOG were included, which may explain the discrepancy between our and these earlier results^42^. The fractalkine/Aβ42 ratio was weakly correlated with PD severity in cross-sectional and longitudinal PD samples^42^. The reason for this may include assay differences, pre-analytical processing, and freeze-thawing effects. In another study, exosomal levels of fractalkine mRNAs were shown to be lower in the CSF of PD patients compared to HC^43^.

The main limitation of this study was the small sample size. Nevertheless, this was an exploratory attempt to examine CSF markers in advanced PD patients with FOG. Further, we have added additional analyses that would address the issue of sample size. To address the concerns of potential non-normality due to limited sample size, we have compared the initial results to those of a nonparametric “ANOVA”—and shown that the results remained the same. We also added a latent variable analysis which supported the primary findings. Another limitation was the cross-sectional nature. Larger and longitudinal studies to examine the trajectory of change of fractalkine and Aβ42 markers will be important in attempting to replicate these findings.

In conclusion, we examined AD and inflammatory-related CSF markers in a small sample of advanced PD patients with and without FOG. We found high levels of Aβ42 in PD-FOG and cross-sectional data which may support an increase over time from early to advanced state in the PD-FOG groups specifically. Longitudinal studies are needed to confirm this. Such results support a previously reported role of Aβ42 in the development of FOG. We also found low levels of fractalkine which might suggest an anti-inflammatory effect. This is the first time an association between fractalkine and FOG has been shown. Whether these changes are specific to FOG or relate to the cognitive change often associated with FOG or simply progression of disease requires further exploration.

## Methods

### Participants

The study population included PD patients with FOG (PD-FOG; *N* = 12), without FOG (PD-NoFOG; *N* = 19), and healthy controls (HC; *N* = 12). Clinical features of a subset of these participants (all PD-FOG patients and *N* = 3 PD-NoFOG patients) have been presented in a previous report^13^. Data from the remaining participants were from a separate cohort available in laboratory records; all available cases were used. All participants were recruited from the Emory Movement Disorders center, Cognitive Neurology Clinics, community-based aging projects, and the Emory Goizueta Alzheimer Disease Research Center^44^. All subjects provided written informed consent prior to participating in protocols approved by the institutional review board of Emory University.

Inclusion criteria were as follows. HC participants: Age ≥18 years; no neurological or orthopedic disorders interfering with gait; no dementia or other medical problems precluding completion of the study protocol. PD participants: Age ≥18 years; PD diagnosis according to United Kingdom Brain Bank criteria^45^; Hoehn & Yahr stage I-IV in the OFF state; demonstrated response to levodopa; able to sign a consent document and willing to participate in all aspects of the study; no atypical parkinsonism or neurological or orthopedic disorders interfering with gait other than Parkinson’s disease; no dementia or other medical problems precluding completion of the study protocol. To be included in the PD-FOG group, participants met additional inclusion criteria: FOG reported using a standardized questionnaire, confirmed by referring neurologist, and verified visually by the examiner using three-dimensional optical motion capture during a levodopa challenge procedure, described below.

### Evaluation of FOG

Because of the noted inaccuracy of self-reported FOG^46^, we verified the presence of FOG with a multistep process. First, we used question 1 of the New FOG questionnaire (NFOG-Q): “Did you experience “freezing episodes” over the past month?” Second, FOG reported with this question was confirmed visually by a movement disorder neurologist referring the patient during a clinic visit. Finally, FOG was verified using a levodopa challenge paradigm conducted in a motion capture laboratory with a three-dimensional optical motion capture system (Motion Analysis Corporation, Santa Rosa, CA) using a superset of the standard Helen-Hayes marker set^47^. The motion capture room measures 5.8 m × 9.0 m with a capture area of 3.0 m × 4.6 m. It is equipped with 14 “Osprey” cameras with a resolution of 640 × 480 running at 120 hz. An example of the user interface is depicted in Supplementary Fig. 1.

During the levodopa challenge paradigm, patients wore tight-fitting clothes and were instrumented with reflective adhesive markers as recommended by the motion capture system manufacturer. They performed a battery of standardized tasks, including Timed Up & Go (TUG) with cognitive and manual dual tasks^48^, in both the OFF and ON medication states. Motion capture recordings of each TUG were reviewed by a movement disorder neurologist (SAF) to score FOG severity according to MDS-UPDRS-III criteria^13,49^. Patients who demonstrated FOG episodes in any of the testing conditions were classified as PD-FOG.

### CSF analysis

CSF (20 mL) was collected using protocols modified from the Alzheimer’s Disease Neuroimaging Initiative (ADNI)^50^ using 24 G Sprotte atraumatic needles and syringe between 8 AM and noon without overnight fasting and transferred into two 15 mL polypropylene tubes. CSF was immediately aliquoted (500 μL), labeled, and frozen (−80 °C) until analysis (Fujirebio, Ghent, Belgium)^44^.

Established CSF Alzheimer’s disease (AD) markers (Aβ42, total tau [t-Tau], and tau phosphorylated at threonine 181 [p-Tau_181_]) were measured using AlzBio3 assays (Fujirebio Diagnostics Inc., Malvern, PA) in a Luminex 200 platform^51^. In addition, ten inflammation-related proteins were selected for their preferential association with innate immunity or different immune cell populations: proinflammation cytokines included tumor necrosis alpha (TNFα), interleukin 7 (IL-7), interleukin 8 (IL-8), transforming growth factor-alpha (TGFα), interferon gamma-induced protein 10 (IP-10), monocyte chemoattractant protein 1 (MCP-1); anti-inflammatory proteins included macrophage-derived chemokine (MDC), interleukin 9 (IL-9), interleukin 10 (IL-10), and fractalkine. All biomarkers analyzed are summarized in Supplementary Table 1. All these proteins were measured in a Luminex 200 platform using the Merck-Milliplex MAP Human Cytokine Panel (HCYTOMAG-60K, Merck-Millipore, Burlington, MA) following the manufacturer’s protocol. All operators were blinded to the diagnosis^44^. In our laboratory, we achieve average intermediate precision (over experiments performed over 9 days) of 9.4% for TNF-α, 12.9% for MDC, 14.7% for IL-7, 4.8% for IP-10, 12.0% for IL-10, 9.2% for IL-9, and 7.6% for IL-8.

### Statistical analysis

Differences in demographic and clinical variables across groups were assessed with tests of central tendency (chi-squared, ANOVA). Differences in clinical variables between the PD-FOG and PD-NoFOG groups were assessed with *t*-tests. All statistical tests were performed in R software at α = 0.05. Procedures used to control family-wise error rate are described below.

### Variation in biomarker expression across groups

Crude differences in average levels of 14 putative CSF biomarkers (Supplementary Table 1) across study groups were assessed with separate one-way ANOVAs followed by multivariate linear models (*lm* in R software). Biomarkers that survived initial ANOVAs after adjustment for false discovery rate with a Benjamini–Hochberg procedure^52^ (*stats::p.adjust*) were entered into separate multivariate linear models with factors coding for presence of PD, presence of FOG, female sex, and PD duration. PD duration in years was centered about 0 prior to entry in linear models such that HC were coded with a value of 0, PD patients with above-average PD duration were coded with a positive number, and PD patients with below-average PD duration were coded with a negative number. Statistical significance of linear model terms for PD and FOG in multivariate linear models was determined with Wald tests, with resulting *P* values once again adjusted for false discovery rate. Additional *F*-tests was applied post hoc to compare the PD-NoFOG and PD-FOG groups while controlling for sex and duration. To guard against non-normality due to sample size limitations, *P* values from one-way ANOVAs were compared to those from Kruskal–Wallis tests. To guard against confounding by sex, *P* values from one-way ANOVAs were compared to those from ANOVAs conducted only among males. Additional details of statistical tests are reported in Supplementary Information.

### Variation with disease duration

Associations between expression of biomarkers that varied significantly with either presence of PD or presence of FOG and PD duration were examined with separate linear regression models with terms for disease duration, age, and female sex. Models were coded with the PD-NoFOG group treated as the reference group. Statistical significance of FOG•duration interaction terms was determined with Wald *t*-tests (*lm* in R software).

### Discriminant partial least squares regression

Discriminant partial least squares regression (D-PLSR) analysis identifies axes, called latent variables (LVs), which consist of profiles of analytes that separate samples based on discrete grouping variables. We performed D-PLSR analysis in R using the *ropls* package available on Bioconductor.org. Grouping variables were created for the presence of PD and the presence of FOG; biomarker data were *z*-scored prior to inputting into the algorithm. Orthogonal rotations were applied to the sample scores and analyte weightings to obtain sample separation according to a discrete group along the LV1 axis. Error bars for LV analyte weightings were calculated by iteratively excluding five random samples without replacement 100 times and regenerating the D-PLSR model each time. Error bars in the LV plots report the mean and standard deviation (SD) computed across the D-PLSR models generated to provide an indication of the variability within each analyte among models. Differences in LV1 expression across groups were assessed with ANOVA (*stats::anova*) followed by post hoc tests with Bonferroni–Holm correction (*stats::pairwise.t.test*).

### Reporting Summary

Further information on research design is available in the Nature Research Reporting Summary linked to this article.

## Supplementary information

Supplementary Information

Reporting Summary

## Data Availability

The datasets generated during and/or analyzed during the current study are available from the corresponding author on reasonable request.
