# Supplementary material for: Cerebrospinal fluid biomarkers in Parkinson’s disease with freezing of gait: an exploratory analysis
Source: NPJ Parkinsons Dis. 2021 Nov 29;7:105. doi: 10.1038/s41531-021-00247-x (PMC8629994; doi:10.1038/s41531-021-00247-x)
Supplement: Supplementary file 1 — Supplementary Information [file 41531_2021_247_MOESM1_ESM.pdf]

Cerebrospinal fluid biomarkers in Parkinson's disease with freezing of gait: an exploratory analysis

J.M. Hatcher-Martin, J.L. McKay, A.F. Pybus, B. Sommerfeld

J.C. Howell, F.C. Goldstein, L Wood, W.T. Hu, S.A. Factor

Supplementary Information

Supplementary Section 1. Candidate biomarkers entered into analyses

Candidate CSF markers entered into analyses were classified as AD-related, pro-, or anti-inflammatory. They are summarized in **Supplementary Table 1**.

Supplementary Table 1. Candidate markers entered into analyses.

Description	Marker
Alzheimer's disease-related	Aβ42
	t-tau
	p-tau
Pro-inflammatory	TGFα
	IL-7
	TNFα
	IL-8
	IP-10
	MCP-1
Anti-inflammatory	MDC
	Fractalkine
	IL-9
	IL-10

Supplementary Section 2. Univariate test results

Expression of CSF biomarkers across groups and results of univariate ANOVAs are summarized in **Supplementary Table 2**. P values from independent one-way ANOVA's applied to each biomarker are summarized in column 5.

To protect against violations of normality due to the small sample size, and to potential confounding by sex, P values from initial ANOVAs were compared to those 1) from non-parametric Kruskal-Wallis tests applied post-hoc (column 6), and 2) from stratified ANOVAs conducted only among males (column 7). In both cases, P values for **Aβ42**, **p-Tau$_{181}$**, and **fractalkine** remained statistically-significant. This provides evidence that the primary results of the paper do not result exclusively from the relatively small sample size and do not exclusively reflect sex imbalances between groups.

Supplementary Table 2. Expression of all CSF biomarkers across study groups. P values from ANOVA, non-parametric Kruskal-Wallis tests, and ANOVA among only the male stratum are presented for comparison.

Biomarker	HC (N=12)	PD-NoFOG (N=19)	PD-FOG (N=12)	P value (ANOVA)	P value (Kruskal-Wallis)	P value (ANOVA among males)
**Ab42**				< 0.01	0.01	0.01
Mean (SD)	250.6 (88.3)	198.9 (72.8)[a]	354.7 (137.3)			
**tTau**				0.58	0.21	0.45
Mean (SD)	36.7 (19.0)	30.5 (17.9)[a]	36.8 (20.0)			
**pTau181**				< 0.01	<0.01	<0.05
Mean (SD)	24.1 (7.8)	15.7 (9.4) [a]	12.9 (5.0)			
**tTauAb42ratio**				0.40	0.27	0.24
Mean (SD)	0.2 (0.1)	0.2 (0.1) [a]	0.1 (0.1)			
**TGFalpha**				0.05	0.06	0.03
Mean (SD)	9.6 (1.7)	8.2 (1.9)	7.9 (1.5)[b]			
**Fractalkine**				< 0.01	<0.01	<0.01
Mean (SD)	67.5 (11.3)	59.0 (7.8)	44.9 (17.3)			
**IL7**				0.78	0.26	0.80
Mean (SD)	4.3 (1.6)	3.9 (2.3)	5.3 (9.6)			
**IL8**				0.93	0.99	0.69
Mean (SD)	60.3 (16.1)	60.1 (17.5)	62.6 (21.5)			
**IL9**				0.24	0.46	0.41
Mean (SD)	5.5 (2.7)	4.3 (1.7)	4.2 (2.1)			
**IL10**				0.39	0.38	0.58
Mean (SD)	4.2 (2.6)	4.3 (1.3)	5.3 (2.8)[b]			
**MDC**				0.35	0.13	0.65
Mean (SD)	68.4 (25.6)	78.6 (59.8)	98.8 (52.9)[b]			
**IP10**				0.38	0.02	0.63
Mean (SD)	2338.0 (1299.0)	3189.2 (2361.6)	3293.4 (1259.0)[b]			
**MCP1**				0.04	0.05	0.13
Mean (SD)	5460.9 (1087.6)	4751.8 (1092.1)	4324.5 (817.6)[b]			
**TNFalpha**				0.22	0.18	0.29
Mean (SD)	3.2 (0.6)	2.8 (0.9)	2.4 (1.5)			

All values are expressed as [Mean (SD)] pg/ml. P values reflect one-way ANOVA across groups (third-to-last column), Kruskal-Wallis rank-sum tests across groups (second-to-last column), and ANOVA across groups among males only (last column). [a]N=17. [b]N=11.

Supplementary Section 3. Details of multivariate linear models

CSF biomarkers that survived initial univariate ANOVA (Biomarker expression ~ Group) and correction for false discovery rate [*stats::p.adjust(method = "fdr")*] were analyzed with subsequent multivariate linear models that controlled for sex and disease duration. Models were formulated with the following equation:

$$expression = \beta_0 + \beta_{PD} PD + \beta_{FOG} FOG + \beta_{Female} Female + \beta_{Duration} Duration + error$$

Where *PD*, *FOG*, and *Female* are dichotomized dummy variables indicating the presence of PD, FOG, and Female sex, respectively, and *Duration* is the centered and scaled numerical value of PD duration.

We note that because FOG patients are a strict subset of PD patients, the coefficients of this model are interpreted as follows. If the null hypothesis that $\beta_{PD} = 0$ is rejected, there is a significant difference between the HC group and the two groups with PD. If the null hypothesis that $\beta_{FOG} = 0$ is rejected, there is a significant difference between the PD-FOG group and the other two groups. Because there was interest in the specific contrast between the PD-NoFOG and PD-FOG groups for the biomarker Fractalkine, one F-test was run post-hoc only among patients with PD. In this test, if the null hypothesis $\beta_{FOG} = 0$ is rejected, there is a significant difference between PD-NoFOG and PD-FOG groups. The results of this contrast are reported in the main text and in **Figure 1**.

Supplementary Section 4. Details of FOG confirmation in kinematic motion capture data

After establishing that FOG was likely to be present based on responses to the New Freezing of Gait questionnaire (NFOG-Q)[1] and agreement by the referring movement disorder neurologist, we verified that FOG was present using a 3-dimensional optical motion capture system (Motion Analysis Corporation, Santa Rosa, CA) during a standardized behavioral testing session. The motion capture facility is located in our clinical center and measures 5.8 m x 9.0 m with a capture area of 3.0 m x 4.6 m, and is equipped with 14 "Osprey" cameras with a resolution of 640 x 480 running at 120hz (**Supplementary Figure 1A**).

During the behavioral testing session, patients wore tight-fitting clothes and were instrumented with reflective adhesive markers as recommended by the motion capture system manufacturer, configured as a superset of the standard Helen-Hayes marker set.[2] Patients performed a battery of standardized tasks, including Timed Up & Go (TUG) with cognitive and manual dual tasks,[3] in both the OFF and ON medication states.[4]

Motion capture recordings of each TUG were reviewed by a movement disorder neurologist (SAF) to score FOG severity according to MDS-UPDRS-III criteria.[5] Patients who demonstrated FOG episodes in any of the testing conditions were confirmed as PD-FOG; patients who did not demonstrate FOG were classified as PD-NoFOG in analyses. An example of the user interface used for identifying FOG episodes in 3-dimensional motion capture data is depicted in **Supplementary Figure 1B**. As opposed to 2-dimensional video recording, the use of 3-dimensional motion capture allows the identification of FOG episodes without occlusion by body segments.[6]

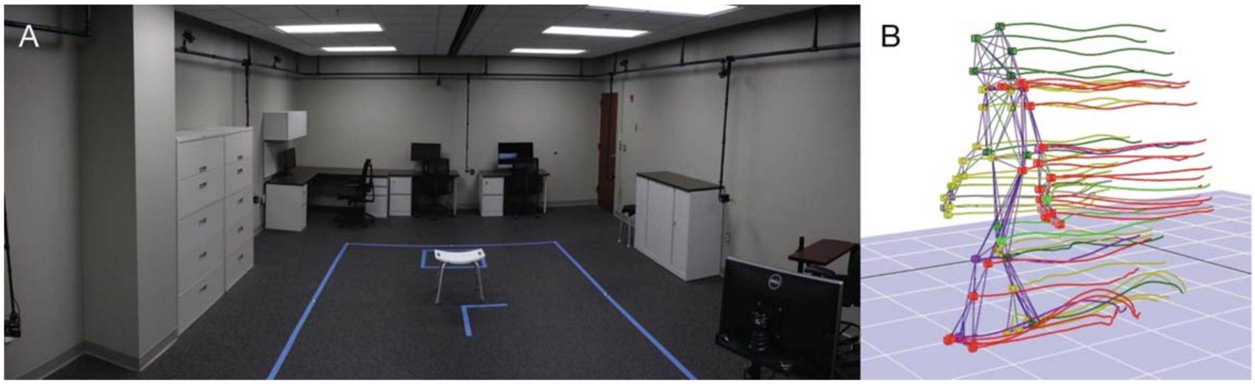

Supplementary Figure 1. Motion capture facility (A) and example of user interface for the 3-dimensional optical motion capture system during a TUG trial (B). Trails from left to right represent the recent positions of each marker.

Supplementary References

1. Nieuwboer A, Rochester L, Herman T, Vandenberghe W, Emil GE, Thomaes T, et al. Reliability of the new freezing of gait questionnaire: agreement between patients with Parkinson's disease and their carers. Gait Posture. 2009;30: 459–63. doi:10.1016/j.gaitpost.2009.07.108

2. Kadaba MP, Ramakrishnan HK, Wootten ME. Measurement of lower extremity kinematics during level walking. J Orthop Res. 1990;8: 383–392. doi:10.1002/jor.1100080310

3. Shumway-Cook A, Baldwin M, Polissar NL, Gruber W. Predicting the probability for falls in community-dwelling older adults. Phys Ther. 1997;77: 812–819.

4. McKay JL, Goldstein FC, Sommerfeld B, Bernhard D, Perez Parra S, Factor SA. Freezing of Gait can persist after an acute levodopa challenge in Parkinson's disease. Npj Park Dis. 2019;5: 25. doi:10.1038/s41531-019-0099-z

5. Goetz CG, Tilley BC, Shaftman SR, Stebbins GT, Fahn S, Martinez-Martin P, et al. Movement Disorder Society-sponsored revision of the Unified Parkinson's Disease Rating Scale (MDS-UPDRS): Scale presentation and clinimetric testing results. Mov Disord. 2008;23: 2129–2170. doi:10.1002/mds.22340

6. Hu K, Wang Z, Wang W, Ehgoetz Martens KA, Wang L, Tan T, et al. Graph Sequence Recurrent Neural Network for Vision-Based Freezing of Gait Detection. IEEE Trans Image Process. 2020;29: 1890–1901. doi:10.1109/TIP.2019.2946469
